# P-561. Geospatial Analysis of Pediatric Patients with Malaria in the Washington Metropolitan Area

**DOI:** 10.1093/ofid/ofaf695.776

**Published:** 2026-01-11

**Authors:** Elijah G Thalos, Shreya Doshi, Anand Gourishankar, Emily Ansusinha, Barbara A Jantausch, Alexandra B Yonts

**Affiliations:** The George Washington University, Arlington, VA; Children's National Health System, Washington DC, DC; Children's National Hospital/George Washington University, Washington, District of Columbia; Children's National Hospital, Washington, District of Columbia; Children's National Hospital, Washington, District of Columbia; Children's National Hospital/ George Washington University, Washington, District of Columbia

## Abstract

**Background:**

In the United States, most malaria cases are related to travel to endemic areas and disproportionately affect specific communities. The burden of malaria and relationship with social determinants of health in developed countries is understudied. Our objective was to report the geographic distribution and any demographic associations of children with malaria in the DC metropolitan area.

**Figure 1:**
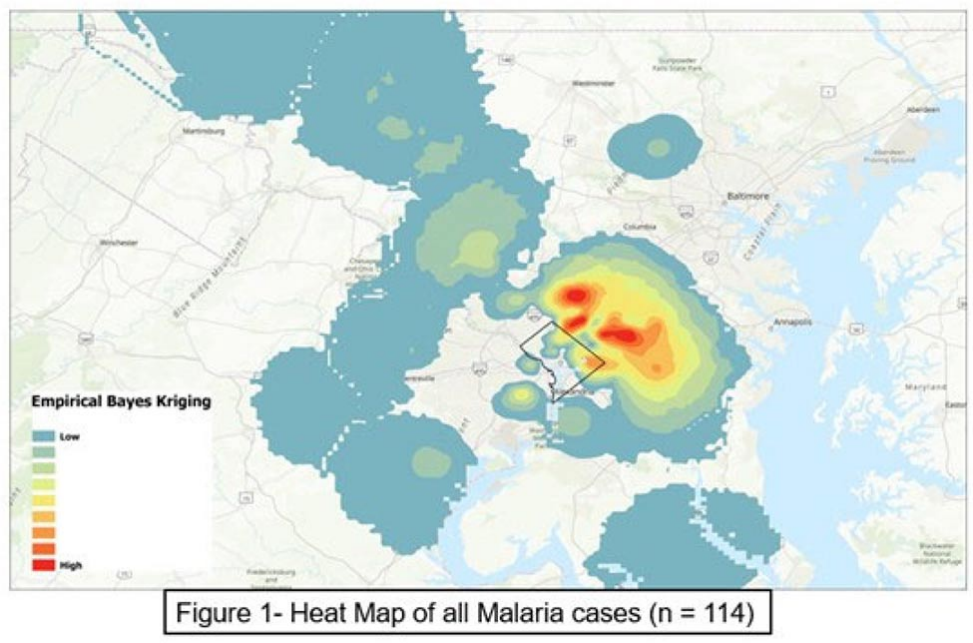
Heat map of all malaria cases (n = 114)

**Figure 2:**
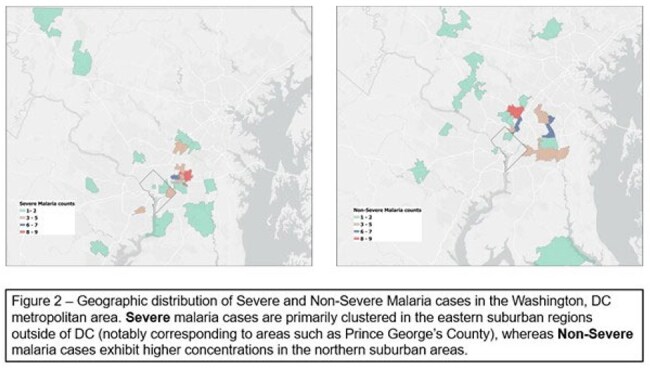
Geographic distribution of Severe and Non-Severe Malaria cases in the Washington, D.C. metropolitan area. Severe malaria cases are primarily clustered in the eastern suburban regions outside of D.C. (notably corresponding to areas such as Prince George's County), whereas Non-Severe malaria cases exhibit higher concentrations in the northern suburb areas.

**Methods:**

We conducted a retrospective study of children and adolescents up to 21 years of age with malaria admitted to a single center from 2018 to 2023. We extracted demographics, clinical, insurance, and travel history data. Using Empirical Bayes Kriging, we created a smoothed map predicting case densities; Choropleth maps and nonparametric test statistics were applied as appropriate.

**Figure 3:**
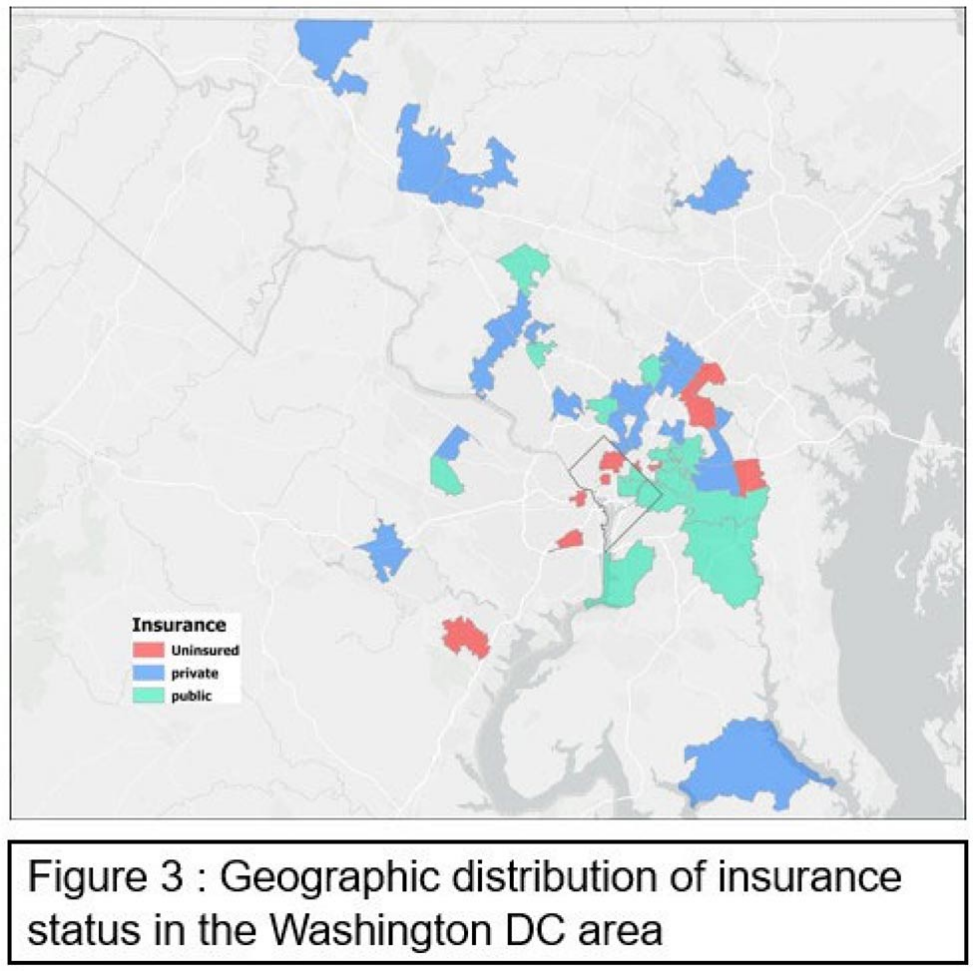
Geographic distribution of insurance status in the Washington D.C. area

**Figure 4:**
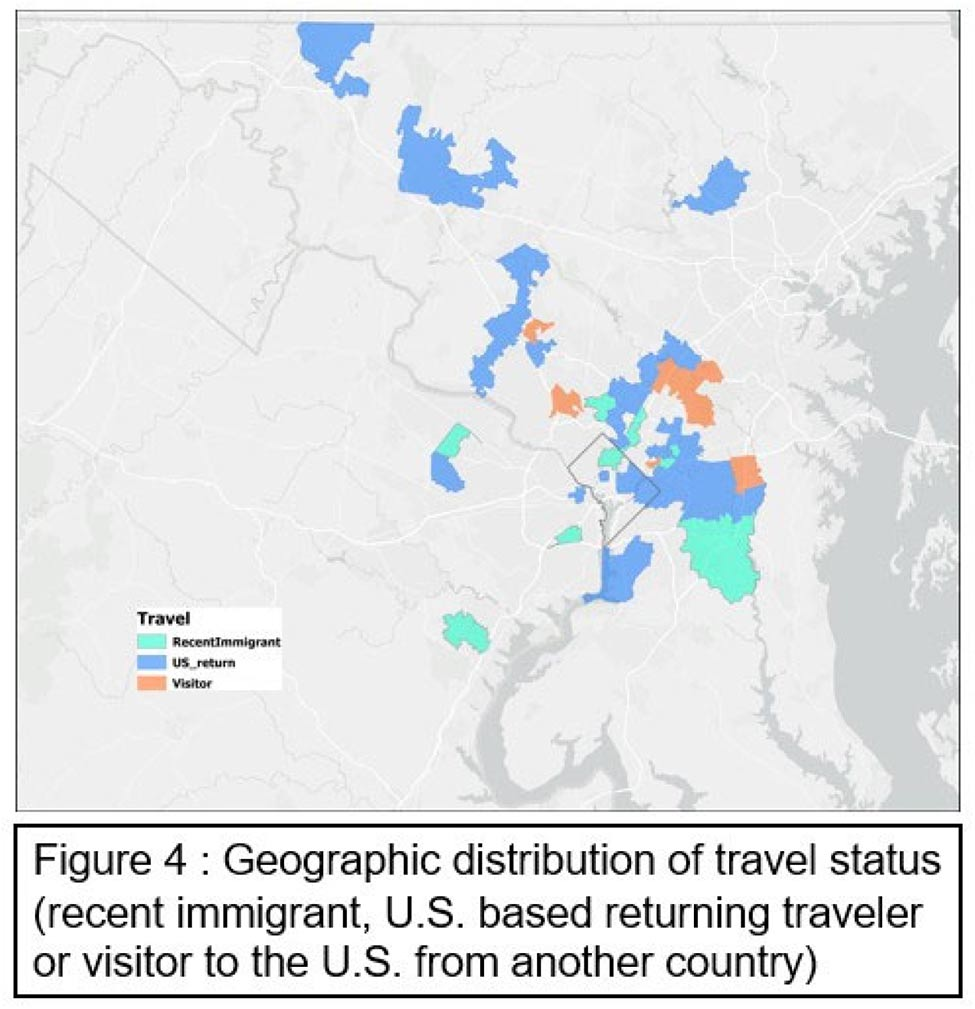
Geographic distribution of travel status (recent immigration, U.S. based returning traveler or visitor to the U.S. from another country)

**Results:**

We identified 114 patients with malaria (65 severe, 49 non-severe). Patients with severe malaria were younger (median age 8.4 years [IQR 4.2–12.8] vs 12.4 [8.7–15.0], p=0.002) than those with non-severe malaria. Sex, race, insurance status did not differ between severe and non-severe malaria. Overall case density was highest in DC and adjacent Maryland (Figure 1). Severe cases were clustered in the Eastern suburbs of DC , whereas non-severe cases were concentrated in the North/Northwestern suburbs (Figure 2). Insurance status is mapped widely, with public insurance densest in Southeast DC and Maryland (Figure 3). Travel-status mapping demonstrated that recent immigrants, U.S.-based returning travelers, and visitors to the U.S. each exhibited unique spatial patterns, with recent immigrants dispersed all over the metropolitan area (Figure 4).

**Conclusion:**

Geospatial mapping of pediatric malaria admissions due to international travel in the DC metropolitan area reveals distinct clusters and patterns. The future includes analysis of clinical severity within a social-determinants-of-health framework to pinpoint vulnerable communities and guide targeted public health interventions. These geospatial insights can direct culturally tailored outreach—such as pre-travel counseling in identified hotspots—and inform policies on equitable access to prophylaxis and care.

**Disclosures:**

Alexandra B. Yonts, MD, Pfizer: Grant/Research Support

